# Voltage-Gated Sodium Channels: A Therapeutic Target in Ischemic Heart Disease

**DOI:** 10.31083/RCM27140

**Published:** 2025-06-26

**Authors:** Xiao-Lu Zhang, Tian-Peng Wei, Fan Yang, Huan-Huan Liu, Ling-Ling Qian, Ru-Xing Wang

**Affiliations:** ^1^Department of Cardiology, Wuxi People’s Hospital Affiliated to Nanjing Medical University, 214023 Wuxi, Jiangsu, China; ^2^Wuxi School of Medicine, Jiangnan University, 214122 Wuxi, Jiangsu, China

**Keywords:** Nav1.5, myocardial infarction, arrhythmia, *SCN5A*

## Abstract

Myocardial infarction (MI)-related arrhythmias are an essential risk factor in sudden cardiac death. Aberrant cardiac the cardiac voltage-gated sodium channel (Nav1.5) is important in the development of ventricular arrhythmias after an MI. These mechanisms are profoundly complex and involve sodium voltage-gated channel α subunit 5 (*SCN5A*) and sodium voltage-gated channel α subunit 10 (*SCN10A*) single nucleotide polymorphisms, aberrant splicing of *SCN5A* mRNAs, transcriptional and post-transcriptional regulation, translation, post-translational transport, and modification, along with protein degradation. These mechanisms ultimately promote a decrease in peak sodium currents, an increase in late sodium currents, and changes in sodium channel kinetics. This review aimed to explore the specific mechanisms of Nav1.5 in post-MI arrhythmias and summarize the potential of therapeutic drugs. An in-depth study of the effect of Nav1.5 on arrhythmias after myocardial ischemia is of crucial clinical significance.

## 1. Introduction

**Fig. 1.**
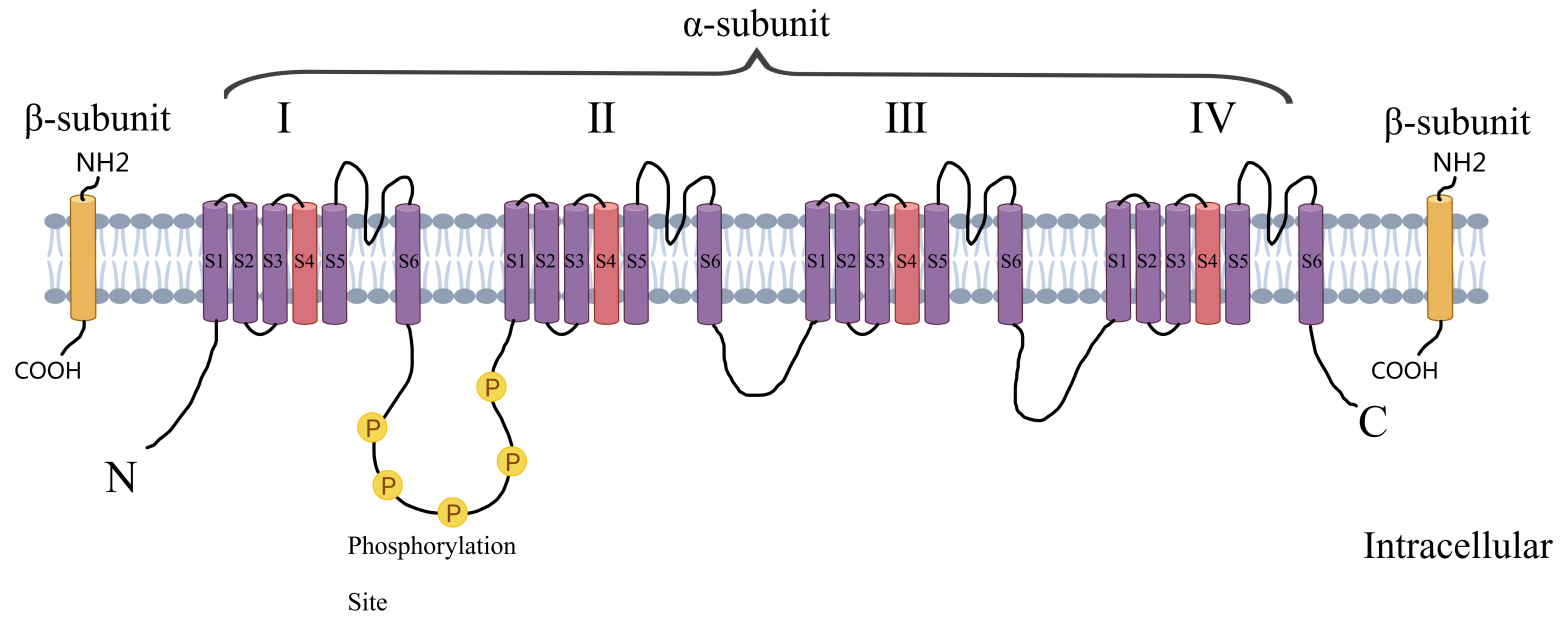
**Structure of voltage-gated sodium channels**. Created by MedPeer (v3.0) (Beijing MedPeer Technology Co., Ltd., Beijing, China).

Myocardial infarction (MI) is one of the most common causes of hospitalization and death in cardiovascular diseases. Post-MI arrhythmias are at the forefront of the risk factors for sudden cardiac death (SCD). Both structural remodeling and electrocardiographic remodeling are essential mechanisms in the development of arrhythmias after an MI. Molecular changes are the main basis for abnormalities in ion channel expression and function, causing posterior depolarization ultimately leading to arrhythmias. The cardiac voltage-gated sodium channel (Nav1.5) is responsible for the rapid, initial upstroke of the action potential and is therefore a key determinant of cardiomyocyte excitability and conduction of electrical impulses through the myocardium [1]. It consists of a cytoplasmic N-terminus, four transmembrane structural domains connected by intracellular and extracellular loops (DI-DIV), as well as a cytoplasmic C-terminal structural domain. Each homologous DI-DIV structural domain comprises six fragments (S1–S6), where S5 and S6 form an ion-conducting channel pore and the highly charged S4 fragment acts as a voltage sensor (Fig. 1). Numerous studies [2, 3, 4] have pointed to an increased incidence of arrhythmias after myocardial ischemia, which is associated with abnormal cardiac Nav1.5 expression or function. The regulation of Nav1.5 after myocardial ischemia is very complex. Through in-depth analysis of its genetic variation, signaling regulation and drug intervention strategies, it is expected that more precise and effective arrhythmia treatment strategies will be realized in the future to reduce the incidence of SCD and improve the quality of survival of MI patients. Therefore, Nav1.5 is not only a core pathological target of post-ischemic arrhythmia, but also provides a new direction for anti-arrhythmic drug development in the era of precision medicine.

The voltage-gated sodium channel consists of a cytoplasmic N-terminal structural domain, four transmembrane structural domains (I–IV) connected by intracellular and extracellular loops, and a cytoplasmic C-terminal structural domain. Each transmembrane structural domain contains 6 segments (S1–S6), of which S5 and S6 form the ion-conducting channel pore and the highly charged S4 segment acts as a voltage sensor.

## 2. Single Nucleotide Polymorphism (SNP) of the sodium voltage-gated channel α subunit 5 (*SCN5A*) Gene and Their Relation With Post-Myocardial Infarction Arrhythmias

Nav1.5 is encoded by the *SCN5A* gene, which is located on chromosome 3p21. *SCN5A* mutations include gain-of-function mutations producing an increase in late sodium current (INaL), loss-of-function mutations responsible for a decrease in peak INa, in addition to mixed phenotypes with both gain-of-function and loss-of-function properties. *SCN5A* mutations give rise to serious, life-threatening cardiac arrhythmia, including sick sinus syndrome, atrial arrhythmias, ventricular arrhythmias (VAs), Brugada syndrome (BrS) and others.

In patients who develop arrhythmias after an acute myocardial infarction (AMI), loss-of-function mutations are more common in *SCN5A* than gain-of-function mutations. For example, the rare *SCN5A* p.A1427S mutation was detected in a study of patients with AMI and malignant arrhythmias following the use of lidocaine. This mutation resulted in a significant reduction in the peak density of sodium currents, manifested as a loss of sodium channel function, and although steady-state inactivation was not significantly altered, the voltage dependence of the peak conductance was shifted in a positive potential direction, thereby amplifying the blocking effect of lidocaine on sodium channels, which may ultimately induce persistent fatal VAs [5]. Another genetic analysis of 19 patients with AMI complicated by ventricular fibrillation (VF) identified the loss-of-function mutation G400A. Although rare in patients with AMI (1/19), this mutation was highly specific (not found in 364 controls) and showed high pathogenicity. G400A alone resulted in a 70.7% reduction in peak current compared with wild type (*p* ≤ 0.001), which was further reduced to 88.4% when accompanied by the histidine to arginine substitution at codon 558 (H558R) polymorphism (*p* ≤ 0.001). This reduction in sodium current density, accelerated inactivation, and altered steady-state voltage-dependent effects all suggest that this mutation may significantly increase the risk of ventricular fibrillation during acute myocardial ischemia [6].

In addition to pathogenic mutations, common polymorphisms in *SCN5A* are associated with changes in electrocardiography (ECG) parameters. For example, the H558R polymorphism and D1819D were both shown to act as factors influencing QTc length in healthy individuals [7]. Furthermore, H558R polymorphisms detected in patients after an MI were associated with increased QT dispersion at minimal and maximal heart rates and prolonged QT intervals before premature ventricular beats [8], although its association with severe arrhythmic events has not reached statistically significant levels. A clear association between H558R and ventricular fibrillation or AMI was also not found in a study of AMI patients of Caucasian origin in Germany [9]. In addition, a synonymous SNP (rs1805126) found in the *SCN5A* coding sequence close to the miR-24 locus showed a regulatory role. The inhibitory effect of miR-24 on *SCN5A* was enhanced in patients harboring the minor allele, leading to loss of function and significant association with low ejection fraction and high mortality in patients with heart failure, especially in the African-American population [10]. Notably, the minor allele frequency (MAF) of this SNP was 33.6% in populations of European descent, which was associated with significantly longer PR intervals (*p* = 3.35 × 10^-7^) and QRS intervals (*p* = 2.69 × 10^-4^), whereas in the Afro-descendant population although the CAF was higher (50.1%), the association was not significant (*p* = 0.12) [11], a difference that may stem from differences across populations.

Additional SNPs further highlight the complexity of *SCN5A*-related electrophysiological effects. For instance, rs7626962 and rs6599230 have been found to be associated with a shortened PR interval [11]. A genome-wide association study further revealed a loss-of-function mutation SNP rs3922844 within *SCN5A* associated with QRS timeframes, in which the C allele was associated with reduced *SCN5A* RNA expression levels, and that reduced Nav1.5 function may lead to a slower rate of ventricular depolarization, which may in turn prolong the QRS interval. This effect was more pronounced in African Americans than in populations of European descent (*p* = 3 × 10^-23^ in African descent compared to *p* = 3 × 10^-16^ in European descent) [12].

A German study sequenced the entire coding region and flanking intronic regions of 46 patients with ventricular fibrillation during acute myocardial infarction (AMI/VF+) and identified two rare variants, 3578G>A (R1193Q) and 4786T>A (F1596I), with a statistically significant difference in the distribution of R1193Q between patients and controls (*p *= 0.046) . This was previously thought to be associated with the long QT syndrome (LQTS), suggesting that it may be a functionally acquired mutation that may lead to persistent sodium currents by affecting sodium channel inactivation; whereas F1596I, although it has been reported in patients with long QT, its exact mechanism is unclear and requires further validation [13]. Similar investigations in diverse populations reveal additional insights. In Chinese patients with malignant VAs after AMI, Wang *et al*. [14] detected four variants located in *SCN5A*, including two missense variants (c.1673A>G and c.3578G>A). In the literature, c.1673A>G has been described as benign, but it has been suggested that this variant may be associated with enhanced sodium channel function (e.g., long QT syndrome type 3), whereas c.3578G>A has been thought to be a clinically pathogenic variant that may be associated with aberrant sodium channel function, although the exact direction of the altered function is not clear. Another rare variant, c.3621C>T, was enriched in VAs (0.72%), but statistical significance has not yet been established (*p* = 0.6308) [14].

Prospective studies further implicate *SCN5A* in arrhythmias. In a Danish prospective case-control study of patients with first ST-segment elevation myocardial infarction (STEMI), a SNP (rs11720524) located in intron 1 of *SCN5A* was identified as potentially affecting sodium channel function through modulation of gene expression, thereby increasing the risk of VF in patients with STEMI [15]. *SCN5A* variants may also play a role in unexplained cardiac deaths. An analysis of 56 autopsy samples with microinfarct changes but no clear cause of death showed that the *SCN5A* p.H445D (rs199473112) missense variant was extremely rare, was not detected in 600 controls, and predicted to be a deleterious variant by computer simulation. This variant is associated with atrial fibrillation (AF) and may promote atrial repolarization dissociation or trigger abnormal activity by reducing sodium current, but the exact type of functional alteration requires further experimental validation [16].

The Nav1.8 channel encoded by the neurotypical sodium channel gene sodium voltage-gated channel α subunit 10 (*SCN10A*) was also found to be associated with arrhythmogenesis. Blockade of Nav1.8 in the ganglionic plexus increases the incidence of VAs in the heart with an AMI [17]. *SCN10A* adjacent to *SCN5A*, is located on human chromosome 3p21-22. The *SCN5A*/*SCN10A* has two enhancers regulated by T-box transcription factor 3 (TBX3) and T-box transcription factor 5 (TBX5). SNP rs6801957 in the *SCN10A* enhancer disrupts TBX3/TBX5 binding and reduces the enhancer’s activity *in vivo* causing a decrease in the expression of *SCN5A* and *SCN10A*, where alterations in the expression of *SCN5A* underlies the observed effects of the SNP in the functioning of the conduction system [18]. *SCN5A*-*SCN10A* association signaling reveals that polymorphisms in genes regulating cardiac conduction can also influence arrhythmia susceptibility [19]. A case-control study of BrS patients from Europe and Japan identified polymorphic loci *SCN10A* rs10428132 and *SCN5A* rs11708996, with the former having a high degree of genetic association and linkage disequilibrium (r^2^ = 0.97) with the possible association with *SCN10A* rs6801957, which together regulate *SCN10A*/*SCN5A* expression, affecting sodium channel function, while the latter independently affects *SCN5A*, both of which are statistically significantly correlated and together lead to QRS prolongation, but the exact functional mechanism needs further experimental verification [19]. In a comparative analysis of Tunisian patients involving MI patients versus healthy controls, these two mutations significantly increased the risk of VA in patients with MI by affecting sodium channel function and regulatory mechanisms [20].

The SNPs of *SCN5A* and *SCN10A* are summarized in Table 1 (Ref. [5, 6, 8, 9, 10, 11, 12, 13, 14, 15, 16, 18, 19, 20]). The multiple stages of cardiac conduction affected by *SCN5A* polymorphisms demonstrates their feasibility and importance as therapeutic targets for arrhythmias, but whether and what kind of relationship exists between individual SNPs and the occurrence of various arrhythmias needs to be clarified and thoroughly investigated.

**Table 1.** **Single nucleotide polymorphism (SNP) of the *SCN5A* gene**.

Mutations	Research community	Outcome	Mutation frequency	Statistical relevance
*SCN5A* p. A1427S [5]	Single patient with AMI and malignant arrhythmia following lidocaine administration	Loss of function, unrelenting lethal VT and VF after lidocaine administration	Not detected in 200 healthy controls and the NHLBI GO Exome database. Frequency <0.01% in 1000 Genomes database	*p * < 0.01 (Peak current density difference); *p * < 0.05 (Voltage-dependent rightward shift of conductance)
*SCN5A*	patients who developed VF during AMI	Loss of function, VT/VF storms	1/19	*p * ≤ 0.001 (Sodium current reduction)
G400A [6]				
*SCN5A* rs1805124 (H558R) [8, 9]	German Caucasian with a history of ST-segment elevation myocardial infarction Patients			*p* = 0.22 (Controls vs AMI); *p* = 1.0 (AMI/VF+ vs AMI/VF-)
	Multi-center genetic cohort predominantly of European ancestry with supplementary African ancestry [11]	PR and QRS shortening	18.4% (Europeans); 22.2% (Africans)	*p* = 6.25 × 10^–⁢4^ (PR); *p* = 5.20 × 10^–⁢3^ (QRS)
*SCN5A*	GRADE cohort [10]	Low ejection fraction and high mortality in patients with HF	Minor allele (C) frequency is 45%	-
rs1805126	GRAHF: African American patients with HF [10]		C-equivalent frequency is 70%	
	Europeans [11]	PR and QRS lengthening #	33.6%	*p* = 3.35 × 10⁻⁷ (PR); *p* = 2.69 × 10^–⁢4^ (QRS)
	Africans [11]	-	50.1%	*p* = 0.12
*SCN5A*	Africans	PR shortening	5.1%	*p *= 2.82 × 10^–⁢3^
rs7626962 (S1102Y) [11]				
*SCN5A*	Europeans	PR shortening	21.9%	*p* = 2.67 × 10^–⁢5^
rs6599230 (A29A) [11]				
*SCN5A* 4786T>A (F1596I) [13]	German patients with AMI/VF+	Nav1.5 dysfunction and VF	AMI 2.17% (1/46) Controls 0% (0/480)	*p * > 0.05
*SCN5A* 3578G>A (R1193Q) [13]	German patients with AMI/VF+	Nav1.5 dysfunction and VF; LQTS	AMI 2.17% (1/46); Controls 0.2% (1/480)	*p* = 0.046
*SCN5A* rs184934308 3c.3621C>T [14]	Chinese population of patients with malignant VAs following AMI	-	AMI/VA+ 0.72%; AMI 0.45%	*p* = 0.6308
*SCN5A*	African Americans	Loss of function; PR interval variants	0.41	*p* = 3 × 10^–⁢23^
rs3922844 [12]	Europeans		-	*p* = 3 × 10^–⁢16^
	African American	QRS lengthening	0.42	*p* = 4 × 10^–⁢14^
*SCN5A* rs11720524 [15]	Danish patients with STEMI	VF	VF patients: 0.64,	*p* = 0.032
			Non-VF patients: 0.58	
*SCN5A* rs199473112 (p.H445D) [16]	Autopsy samples with myocardial microinfarction changes but no clear cause of death	-	with a frequency of 0.005257% in the ExAC database and not detected in 600 control alleles.	-
*SCN5A* rs11708996	BrS patients in Europe and Japan [19]	QRS lengthening	BrS 0.23; Controls 0.15	*p* = 1.02 × 10^–⁢14^
	Tunisian MI patients [20]	VA	MI 0.27; Controls 0.15	*p* = 0.001
*SCN10A* rs10428132	BrS patients in Europe and Japan [19]	Prolonged QRS interval	BrS 0.69; Controls 0.41	*p* = 1.01 × 10^–⁢68^
	Tunisian MI patients [20]	VA	MI 0.21; Controls 0.10	*p* = 0.001
*SCN10A* rs6801957 [18]	-	decrease in the expression of *SCN5A* and *SCN10A*	-	-

VT, ventricular tachycardia; VF, ventricular fibrillation; NHLBI, national heart, lung, and blood institute; AMI, acute myocardial infarction; MI, myocardial infarction; STEMI, first ST-segment elevation myocardial infarction; HF, heart failure; BrS, Brugada syndrome; PR, pulmonary regurgitation; GRADE, grading of recommendations assessment, development and evaluation; GRAHF, global registry of acute heart failure; VA, ventricular arrhythmia; ExAC, Exome Aggregation Consortium. #, currently controversial.

## 3. Alteration of Nav1.5 after Myocardial Infarction

### 3.1 Sodium Channel Expression

The morphology and function of cardiac sodium channels begins to remodel within hours after coronary artery occlusion, and this change occurred as early as 3 hours following coronary artery occlusion in dogs, with a decrease in Nav1.5 protein expression in the epicardial marginal zone found by 48 hours after infarction, and a significant decrease in Nav1.5 protein of 42% 5 days after occlusion [21]. The expression of Nav1.5 was similarly reduced in the infarct border zone of mice 1 week after an MI [2]. By 5 weeks after infarction, the level of Nav1.5 protein expression in sprague-dawley (SD) rats remained low and was 38% lower than in the sham-operated group [22, 23]. The protein level of Nav1.5 in heart tissue of patients with end-stage Ischemic Heart Disease (IHD) is decreased, which is the basis of arrhythmia in patients with IHD [2]. Results from both animal models and human tissues suggest that clarifying the specific mechanism of reduced Nav1.5 expression after MI is important for post-infarction arrhythmia prevention and treatment.

The sodium channel β-subunit performs an auxiliary role to the α-subunit, and its function is currently uncertain. In a canine model of heart failure established by multiple sequential coronary artery microsphere embolizations the protein levels of Navβ1 and Navβ2 subunits remained unchanged, but reduced protein levels of Nav1.5 caused a relative upregulation of these β-subunits, and this change caused an alteration of the INaL, in which knockdown of Navβ1 reduced the density of the INaL, and Navβ2 and Navβ1 had opposite effects on INaL regulation [24]. This indicates that the Na^+^ channel β-subunit may also play a key role in maintaining the normal function of voltage-gated channels as well as abnormal changes in pathological states. Variations in Navβ1 and Navβ3 affect the disease phenotypes of AF and BrS by affecting channel expression and directly affecting channel gating [25]. In the presence of lidocaine and ranolazine, Navβ1 enhanced the modulation of the structural domain DIII-VSD by lidocaine but inhibited the effects of ranolazine; In contrast, Navβ3 eliminated the modulatory effects of lidocaine but enhanced the effects of ranolazine on DIII-VSD. Alteration of subunits expression will have a predictable impact on therapeutic efficacy, thereby optimizing the use of lidocaine and ranolazine in arrhythmia therapy [26].

Cardiac ischemia also causes changes in the other sodium channels. For instance, brain subtype NaCh I protein expression is increased in remodeled myocardium following an MI, and the NaCh Ia/NaCh I isoform ratio is reversed toward the fetal phenotype [27]. Skeletal muscle NaCh isoform Nav1.4 (previously known as SkM1) has been shown to present in human cardiac tissue. Surviving myocardium expressing Nav1.4 channels 1 week after infarction was found to result in an increase in longitudinal conduction velocity and a decrease in the incidence of induced VAs post-infarction [28]. Apart from voltage-gated sodium channels, acid-sensing ion channel (ASICs) of the epithelial sodium channel family of nonvoltage-gated ion channels also respond to cardiac ischemia. ASIC1a, for example, is activated by acidosis caused by cardiac ischemia, and blocking ASIC1a can protect the heart from ischemic injury [29]. Contrary to ASIC1a, ASIC3 is seen as an instrumental mediator in the perception of ischemic pain in the heart and may exert an active role in myocardial ischemia [30].

### 3.2 Peak Sodium Current and Late Sodium Current

Normally, Nav1.5 is rapidly activated to be put in charge of the depolarization of the action potential, followed by inactivation to allow the repolarization of the action potential, where a small fraction of the sodium channels fail to be inactivated or reactivated to produce a late sodium current that lasts for the entire action potential. Therefore, Nav1.5 is a key determinant of cardiomyocyte excitability and the conduction of electrical impulses through the myocardium. It play an important role not only in the triggering of AP in ventricular myocytes but also, to a lesser extent, in the regulation of APs duration by maintaining the duration of the plateau phase and promoting the repolarization phase.

Due to partial depolarization of ischemic cardiomyocytes, sodium channels are predominantly inactivated. The INa inactivation curve of cardiomyocytes after simulated ischemia is significantly shifted toward hyperpolarization, the rate of inactivation is accelerated, and the recovery from inactivation is slower than normal. The activation curve shifts to the right and the activation process slows down. The combined effect results in a decrease in the amplitude of cardiomyocyte AP and a slowdown in impulse conduction.

**Fig. 2.**
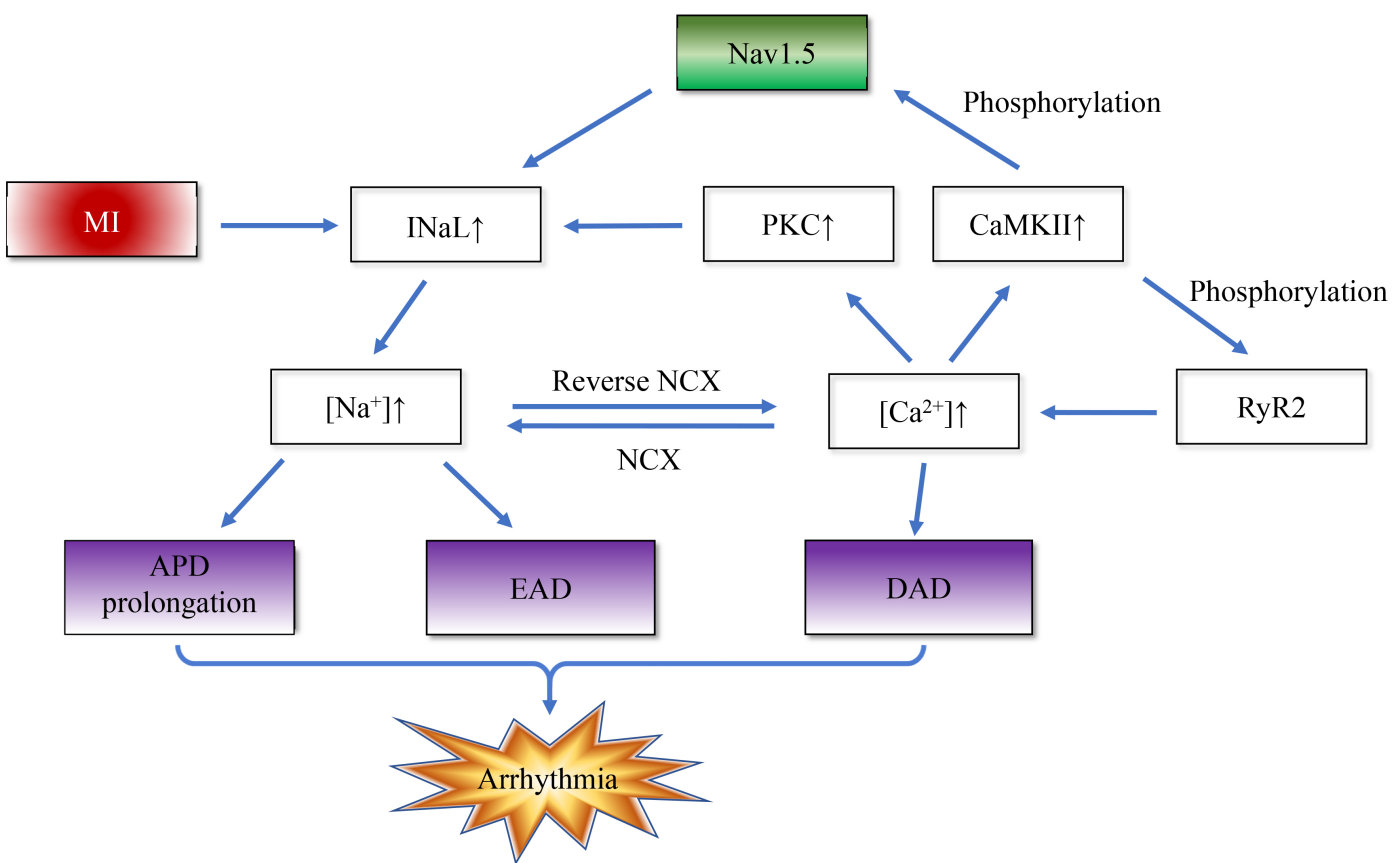
**Myocardial infarction disrupts intracellular sodium/calcium homeostasis and positive feedback**. The enhanced late sodium current (INaL) after MI induces arrhythmias through a bidirectional positive feedback mechanism: On the one hand, enhanced INaL leads to increased sodium influx, triggering calcium overload through the Na^+^/Ca^2^^+^ exchanger (NCX). The activated calmodulin (CaM) / calcium/calmodulin-dependent protein kinase II (CaMKII) complex and protein kinase C (PKC) further amplify INaL by phosphorylating and delaying the inactivation of Nav1.5 sodium channel, respectively. On the other hand, CaMKII phosphorylates ryanodine receptor type 2 (RyR2) to enhance calcium release, and cooperates with NCX to form a “calcium-sodium-calcium” cycle, which ultimately increases arrhythmia susceptibility by prolonging action potential duration (APD) and reducing repolarization reserve. EAD, early afterdepolarization; DAD, delayed afterdepolarization.

As mentioned earlier, congenital and acquired dysfunctions of sodium channel function are known to predispose to a reduction in INa. In contrast to INa, the INaL amplitude is much smaller, but as it flows throughout the action potential, it makes a significant contribution to the sodium load during each cardiac cycle. In pathological states such as myocardial ischemia, metabolites such as palmitoyl-l-carnitine, lysophosphatidylcholine, and reactive oxygen/nitrogen species accumulate and cause INaL enhancement, which can provoke arrhythmias via two mechanisms. The increase in inward current, action potential duration (APD) is prolonged, and cardiomyocyte repolarization reserve is decreased, thereby causing severe arrhythmias and even sudden death when the heart encounters weak arrhythmogenic factors. Enhanced INaL causes sodium channels to open abnormally during the duration of the action potential, leading to excessive inward flow of sodium ions. In addition, energy supply is limited during ischemia, and the efficiency of the sodium-potassium pump (Na^+^/K^+^-ATPase) is reduced, leading to intracellular Na^+^ accumulation [31, 32]. Intracellular calcium overload from decreased Ca^2+^ ion efflux through the Na^+^/Ca^2+^ exchanger (NCX) forward mode and/or increased Ca^2+^ ion influx through the NCX reverse mode [33], Ca^2+^ binds to calmodulin (CaM) to form the Ca^2+^/CaM complex to activate calcium/calmodulin-dependent protein kinase II (CaMKII), leading to CaMKII-dependent phosphorylation of the Nav1.5 Ser571 site and enhanced INaL (27% of cases) [34]. Ca^2+^ synergizes with diacylglycerol in the membrane to activate calcium-sensitive protein kinase C (PKC), delay sodium channel inactivation, and increase InaL (62%) [35], leading to an increase in intracellular Na^+^ concentration through the above mechanism [36]. Increased Na^+^ inward flow can in turn lead to activation of CaMKII, and activated CaMKII increases calcium release by phosphorylating ryanodine receptor type 2 (RyR2), and phosphorylates Nav1.5, further promoting sodium inward flow [37]. This positive feedback loop emphasizes the utility of INaL in postischemic arrhythmias. Fig. 2 simply illustrates this positive feedback mechanism.

The INaL during the action potential plateau period reduces the repolarization reserve, causing the membrane potential to remain in the plateau period for a longer period of time and increasing the likelihood of reactivation of the L-type calcium channel (ICaL). When the membrane potential stays in the range of –40 to –20 mV, ICaL reopens to generate inward current, further delaying repolarization and creating early afterdepolarization (EAD). In addition, intracellular calcium overload further activates calcium-sensitive ion channels, such as calcium-dependent nonselective cation channels or calcium-activated potassium channels, generating triggered inward currents that predispose membrane potentials to arrhythmias such as EAD. Calcium overload triggers abnormal calcium release function in the sarcoplasmic reticulum (SR), including the appearance of nonphysiologic spontaneous calcium sparks and calcium waves. These spontaneous calcium releases generate depolarizing inward currents through the NCX, creating a delayed afterdepolarization (DAD). Furthermore, INaL-induced sodium-calcium imbalance and intracellular calcium overload activate calcium-sensitive signaling pathways (CaMKII etc.), which further amplify the abnormal calcium release and enhance the magnitude of DAD. When the amplitude of the DAD reaches the threshold potential, it can trigger action potentials that induce triggered arrhythmias, including premature ventricular contractions and AF. Consequently, targeting INaL modulation has become an important antiarrhythmic strategy after myocardial ischemia, including CaMKII and PKC inhibitors, NAD+ supplementation, oxidative stress inhibitors, and selective INaL blockers (e.g., Ranolazine), interventions that are expected to reduce arrhythmias and improve cardiac events by restoring normal Nav1.5 function and improving the clinical prognosis of these patients.

## 4. Regulation Mechanisms of Nav1.5 After Myocardial Infarction

### 4.1 Wnt/β-Catenin Signaling Pathway

Wnt signaling plays a crucial role in cardiac development but is usually silenced after birth. After an MI, in response to cardiac stress and injury, the Wnt/β-catenin signaling pathway was reactivated, and the activity of Wnt signaling remained close to basal levels on day 3 after an MI (9.44%) but increased significantly on day 7 (14.47%), followed by a further increase on days 14 (22.42%) and 21 (24.98%), and stabilized between days 14 and 21. In terms of spatial distribution, Wnt signaling was upregulated throughout the cardiac range, but was mainly concentrated in the infarct border zone and distal region, while some degree of Wnt activity was also observed in the infarct zone [38]. Mechanistically, when the Wnt pathway is not activated, the cytoplasmic degradation complex (including GSK-3β) promotes the phosphorylation of β-catenin, leading to its degradation via the ubiquitin-proteasome system. When Wnt signaling is activated it prevents the destruction complex from phosphorylating β-catenin. β-catenin accumulates in the cytoplasm and translocates to the nucleus [39]. Notably, the activity of the Wnt/β-catenin signaling pathway changes dynamically during I/R injury, and during the early ischemic phase, Wnt/β-catenin signaling is inhibited, leading to an increase in cardiomyocyte apoptosis. During the ischemic phase, activation of Wnt/β-catenin signaling promotes M1-type macrophage polarization and exacerbates inflammation. During myocardial repair, moderate activation of Wnt/β-catenin signaling is thought to promote cardiac neovascularization and fibroblast proliferation, contributing to tissue repair, but excessive activation may lead to increased fibrosis [40]. Precise modulation targeting Wnt/β-catenin signaling at different pathological stages may therefore be a promising therapeutic strategy.

β-catenin regulates Nav1.5 mainly through the classical Wnt/β-catenin signaling pathway. β-catenin interacts with its nuclear chaperone transcription factor 4 (TCF4) directly binding the *SCN5A* promoter to repress its transcription. β-catenin/TCF4 binds to the –871 bp site of the *SCN5A* repressor TBX3 promoter to activate its expression, thereby inhibiting Na^+^ channels. This indirect inhibitory effect of β-catenin signaling through TBX3 is responsible for only 30% of the β-catenin-induced Nav1.5 reduction [41, 42]. Wang *et al*. [41] found that the TCF4 binding site was at –2803 bp, and was uncertain if it was regulated by the β-catenin signaling pathway, In contrast, Lu *et al*. [42] used CRISPR/Cas9 to induce mutations in the binding site in another study to demonstrate that the TCF4 binding site was located in the proximal region of the *SCN5A* promoter (–621 and –131 bp) and that the binding of β-catenin /TCF4 to these two sites is regulated by the β-catenin signaling pathway. The difference between the two studies may be due to the different experimental techniques. Wang *et al*. [41] activated Wnt/β-catenin signaling via H₂O₂, whereas Lu *et al*. [42] used Wnt3a proteins or GSK-3β inhibitors, and the difference in activation may affect the binding properties of the downstream targets.

The therapeutic targeting of this pathway requires a careful balance between risks and benefits. The Wnt signaling pathway is required for homeostasis in many noncardiac tissues, so the use of Wnt inhibitors in cardiac patients causes significant side effects [42]. In I/R, inhibition of GSK-3β attenuates its induction of apoptosis and is cardioprotective. But inhibition of GSK-3 can acutely affect human cardiac electrophysiology in an arrhythmogenic manner by downregulating Nav1.5 expression. Therefore, frequent monitoring of possible arrhythmogenic effects may be required in clinical trials of targeted therapeutic GSK-3 inhibitors for the treatment of non-cardiac diseases [39, 43], LF3, an inhibitor of β-catenin/TCF4 interaction, has now been identified to upregulate Nav1.5 expression, causing an increase in Na^+^ channel activity in HL-1 cardiomyocytes [44]. This may help to prevent the decline in Nav1.5 expression after an MI, but additional studies are needed to explore the various mechanisms that inhibit β-catenin signaling, ie, whether the target of key molecular interactions should be blocked rather than inhibiting the entire Wnt/β-catenin pathway.

### 4.2 Transcriptional and Post-Transcriptional Regulation

The transcription of the gene encoding *SCN5A* into mRNA is regulated by a variety of factors. After cardiac ischemia, certain molecules such as microRNAs and transcription factors are altered to affect Nav1.5 expression at the transcriptional or post-transcriptional level.

MiR-448 increase during ischemia and interact with the 3′-UTR in *SCN5A* mRNA to induce reductions in *SCN5A* mRNA, protein, and currents. The RNA-binding protein human antigen R (HuR) stabilizes mRNA by binding to AU-rich elements (AREs) within the 3′-UTR of *SCN5A* mRNA. By occupying the ARE regions, HuR may hinder the binding of miR-448 to *SCN5A* mRNA. Additionally, HuR promotes *SCN5A* expression by reducing the degradation of myocyte-specific enhancer factor 2C (MEF2C) mRNA transcripts, thereby maintaining the stability of *SCN5A* [3]. Furthermore, bioinformatic analysis shows that let-7f microRNA and MiR-378 expression is upregulated in MI and targets *SCN5A*/Nav1.5, causing a decrease in INa [45]. MicroRNAs also exert an active role in ischemic arrhythmias. MiR-143, an upstream of early growth response protein 1 (EGR1) can mediate Fibroblast growth factor 21 (FGF21)-induced upregulation of EGR1 in cardiomyocytes. Recruitment of EGR1 to the *SCN5A* and KCNJ2 promoter regions results in upregulation of Nav1.5 and Kir2.1 expression at the transcriptional level, providing a novel therapeutic strategy for clinical ischemic arrhythmias [46].

A number of transcription factors have been reputed to regulate the expression of *SCN5A*. The JASPAR database predicts that the *SCN5A* promoter region contains multiple highly conserved Meis1 binding sites. Chromatin immunoprecipitation (ChIP) experiments further confirmed that Meis1 was able to bind to the *SCN5A* promoter region and that Meis1 binding to the *SCN5A* promoter was significantly reduced after an MI. Overexpression of Meis1 restored the expression of Nav1.5 in cardiomyocytes after an MI. Upregulation of CDC20 expression in ischemic cardiomyocytes after an MI leads to accelerated degradation of Meis1 in a ubiquitin-proteasome system, resulting in myocardial sodium channel dysfunction. Overexpression of Meis1 did not revert the downregulation of the infarcted cardiac connexin 43 (CX43) protein, suggesting that the salvage effect of Meis1 on infarcted cardiac conduction is more specific to ion channels than to gap junctions. TBX5, pivotal in cardiac development, regulates the mature cardiac conduction system (especially the ventricular conduction system), in part through direct regulation of an enhancer downstream of *SCN5A* [47]. TBX3, genomically distinct from TBX5, is expressed throughout the central cardiac conduction system and potently inhibits intercellular conduction, INa, and IK1 [48]. The transcription factor Forkhead box protein O1 (FoxO1) accumulates in the nucleus of cardiomyocytes from chronically ischemic human hearts and negatively regulates Nav1.5 expression by altering the promoter activity of *SCN5A* [49]. Transcription factor enhancer of zeste homolog 2 (EZH2), the catalytic subunit of polycomb repressive complex 2 (PRC2), promotes heterochromatin formation by catalyzing H3K27me3, resulting in transcriptional repression [50]. Increased expression of EZH2 and H3K27me3 with enrichment in the *Scn5a* promoter region after cardiac ischemia inhibits the promoter activity of *SCN5A*, leading to decreased Nav1.5 expression and Na^+^ channel activity, which underlie the development of cardiac arrhythmias. Application of the selective EZH2 inhibitor GSK126 significantly increased Na^+^ channel activity in HL-1 cardiomyocytes. Therefore, it could be a feasible antiarrhythmic target against EZH2 for the treatment of IHD [2].

After the mRNA has been transcribed and post-transcriptionally modified to mature mRNA, it passes through the nuclear pore into the cytoplasm where it binds to ribosomes to begin the subsequent translation process. The nuclear pore protein Nup107 promotes the export of mature *SCN5A* mRNA from the nucleus to the cytoplasm in a post-transcriptional manner specifically regulating the distribution of *SCN5A* mRNA, required for subsequent translation and normal cardiac bioelectricity. Nup107 is increased as a fast-acting protein in cardiomyocytes and rat models of infarction during hypoxia and oxidative stress, forcing an increase in the protein level of Nav1.5 in the early stages of hypoxia, but with the prolonged duration of hypoxia existing *SCN5A* mRNA libraries are depleted, and Nav1.5 expression declines [51]. Other nuclear pore proteins also undergo changes following cardiac ischemia [51, 52]. Therefore nuclear pore proteins merit in-depth investigation as novel molecular targets to ameliorate myocardial ischemic injury.

After myocardial ischemia, *SCN5A* gene expression is regulated by multiple mechanisms including microRNAs, transcription factors and nuclear pore proteins. These include microRNA-mediated post-transcriptional regulation, transcription factor regulation and nuclear pore protein-mediated mRNA export, targeting microRNA/RNA-binding protein regulatory networks (e.g., inhibition of miR-448 or enhancement of HuR), interfering with the function of transcription factors (e.g., Meis1 agonists, EZH2 inhibitors), modulating nuclear pore protein-mediated mRNA transport, or enhancing ion channel transcription via TBX5/EGR1 to enhance ion channel transcription. These mechanisms provide new directions for the development of specific anti-ischemic arrhythmia drugs and myocardial protection strategies.

### 4.3 Translation and Post-Translation Modification Regulation 

Mitogen-activated protein kinases (MAPKs) are rapidly activated during myocardial ischemia.This activation occurs not only in the ischemic region of the myocardium, but also in the nonischemic region of the heart [53]. MAPKs are a ubiquitous group of protein serine/threonine kinases acting as crucial mediators of signal transduction pathways responsible for cell growth. This regulatory mechanism allows cells to respond and ultimately adapt to changes in myocardial ischemia. c-Jun N-terminal kinases (JNKs), p38-MAPK (s) and ERKs are expressed in human heart. JNK and p38-MAPK (s) are capable of promoting pathologic cardiac remodeling after an MI [54]. Constitutively phosphorylated/activated ERKs destabilizes Na^+^ channel α-subunit mRNAs through translational events, thereby negatively regulating homeostatic levels of α-subunit mRNAs and cell surface expression of functional Na^+^ channels [55]. Consequently, MAPKs activation during myocardial ischemia leads to arrhythmias by interfering with the translation process of Nav1.5 channels [56].

NAD is a central metabolite involved in energy and redox homeostasis as well as DNA repair and protein deacetylation reactions. Cardiac myocytes accumulate the oxidized form of NAD, NAD+, predominantly in the mitochondria of cardiac myocytes [57]. Cardiac NAD+ levels decline and its reduced form, NAD(P)H, is elevated after I/R in mice. Both of these changes may lead to reduced INa and increased the risk of arrhythmias in the ischemic [58]. Decreased NAD+ levels reduce NAD+-dependent deacetylation of sirtuin 1 (Sirt1) deacetylase. Nav1.5 hyperacetylation on cardiomyocyte membranes induces reduced inotropic expression of Nav1.5 channels, giving rise to cardiac conduction abnormalities and premature death due to cardiac arrhythmias [59]. NADH triggers PKC activation by boosting phospholipase D (PLD) activity. PKC (especially isoform PKCδ) can induce phosphorylation of the Nav1.5 channel at the S1503 locus, either directly, or indirectly by elevating Mitochondrial reactive oxygen species (mitoROS). Phosphorylation of the S1503 site in concert with mitoROS leads to a decrease in the single-channel conductance of the sodium channel, which is the primary mechanism causing the decrease in sodium current, as opposed to a change in the number of channels [58]. NAD+ supplementation protects the heart during ischemia and subsequent reperfusion injury [57]. NAD+ antagonizes the reduction in INA associated with elevated NADH levels by activating protein kinase A (PKA) and reducing the production of mitoROS [60]. Activation of PKA enhances the transport of Na^+^ channels toward the plasma membrane thereby generating an increase in the amplitude of Na^+^ currents [61], while PKC activation promotes the movement of Na^+^ channels away from the cell surface [62]. Therefore, cardiac ischemia also affects INa expression by influencing the transport of Na^+^ channels to the plasma membrane.

Post-translational modification of Nav1.5 is fundamental to the regulation of cardiac Na^+^ channels. AMI results in extensive oxidative damage, and HL-1 cell exposure to oxidants induces lipid oxidation modification of Nav1.5 [63]. Cardiomyocytes secrete mitochondria-derived ROS during ischemia, and ROS-induced ROS release may amplify their signals. Free radical-mediated lipid peroxidation may affect Na^+^ channels embedded in the membrane lipid bilayer, which may result from the formation of covalent adducts between isoketal (IsoK) formed by the isoprostane pathway of lipid peroxidation and lysyl residues on Na^+^ channels [64]. ROS can activate CaMKII via oxidative phosphorylation. Phosphorylation of the CaMKII-dependent Nav1.5 Ser571 site after I/R in isolated mouse hearts, leads to an increased susceptibility to cardiac arrhythmias [34]. Phosphorylation is the most well-documented post-transcriptional modification in cardiac Nav1.5 channels. Nav1.5 hyperphosphorylation promotes spontaneous arrhythmic events by increasing INAL and subsequent Na^+^/Ca^2+^ overload [34]. During I/R, AMPK phosphorylates Nav1.5 at the threonine (T) 101 site and regulates the interaction between Nav1.5 and the autophagy junction protein microtubule-associated protein 1 light chain 3 (LC3) through exposure of the LC3-interacting region in Nav1.5 adjacent to T101, causing Nav1.5 degradation through autophagy [65].

Along with the autophagy pathway, ubiquitination appears to be an important pathway regulating the degradation of Nav1.5. Modifications in the function of the ubiquitin proteasome system (UPS) are implicated in the pathogenesis of myocardial ischemia. The human genome contains only two E1s, UBE1 and UBA6, each of which is required for Nav1.5 ubiquitination and regulation of Nav1.5 expression levels and cardiac sodium current density. Both may act synergistically during Nav1.5 ubiquitination, a role that is stronger for UBA6 than for UBE1 [66]. Sumo-binding enzyme UBC9 is an E2 enzyme for Nav1.5 ubiquitination, which regulates Nav1.5 expression levels in a SUMOylation-independent manner [67]. UBC9 interacts with the E3 enzyme Nedd4-2 and significantly reduces Nav1.5 expression and INa density [66]. Human leukocyte antigen F-associated transcript 10 (FAT10) of the small ubiquitin-like protein family binds to lysine residues in the C-terminal fragment of Nav1.5 and diminishes the binding of Nav1.5 to Nedd4-2, blocking its degradation by the UPS after an MI and suppresses ischemia-induced VAs [4]. Furthermore, FAT10 stabilizes Caveolin-3 (Cav3) [68], whose increased expression leads to a decrease in cardiomyocyte INaL by reducing S-nitrosylation of Nav1.5, and may also play a role in decreasing ischemia-induced arrhythmias [69]. αB-Crystallin interacts with Nedd4-2 to increase cell surface Nav1.5 expression levels by inhibiting Nav1.5 internalization, thereby increasing INa density [70]. Nedd4-2 binds to the Nav1.5 C-terminal PY-motif (xPPxY) and acts on Nav1.5 by reducing cell surface channel density. The decrease in INa is likely to be caused by an increase in the internalization rate rather than by the degradation of Nav1.5. UBR3 and UBR6 of the E3 component. The N-recognin (UBR) family has been shown to reduce the protein levels of Nav1.5 channels by ubiquitination [71]. Future studies will explore whether UBC9 is the only E2 enzyme and also if there are other E3 enzymes that regulate Nav1.5 ubiquitination. Internalization of ubiquitinated Nav1.5 may involve the endosomal-lysosomal pathway, and thus the ubiquitin-lysosomal pathway is likely to be involved in the degradation of ubiquitinated Nav1.5 as well. Additional future studies are needed to distinguish the roles of the ubiquitin-proteasome pathway and the ubiquitin-lysosome pathway in the ubiquitination of Nav1.5 SUMOylation. Ubiquitination, is a ubiquitin-related transient post-translational modification pathway that catalyzes the binding of small ubiquitin-like modifier proteins to protein lysine residues [72]. Hypoxia induces rapid SUMOylation of the Nav1.5 channel lysine 442 site in human cardiac myocytes derived from pluripotent stem cells, which reopens the Nav1.5 channel in the late action potential, leading to sustained sodium inward flow and elevated cardiac INaL [73]. However, more recent studies have found that in HEK293 cells, and SUMOylation of Nav1.5 at the K442 site had no effect on the INaL, but rather increased the membrane surface channel density by facilitating the localization of Nav1.5 at the cell membrane, significantly increasing peak INa [74]. The differences between the two studies may be due to the use of different cells that were studied and differences in experimental conditions.

Beyond the post-translational modifications mentioned above, it was found that Nav1.5 methylation modifications reciprocally regulate phosphorylation modifications at neighboring sites, and that pathogenic Nav1.5 mutations altering the Nav1.5 methylation-phosphorylation balance can increase the risk of cardiac conduction system pathology. Methylation of Nav1.5 R526, a major post-translational modification of any Nav1.5 arginine or lysine residue, was identified from end-stage HF human heart tissue [75]. Methylation’s dynamic regulatory network and its synergistic effect with phosphorylation in ischemic heart injury therefore need to be analyzed in future studies.

These mechanisms provide multilevel targets for therapeutic development: inhibition of MAPKs (e.g., ERK/p38 antagonists) or CaMKII improves channel stability; supplementation of NAD+ or activation of Sirt1/PKA may restore deacetylation and membrane translocation efficiencies; modulation of the ubiquitination system (e.g., blockade of Nedd4-2, augmentation of FAT10) or inhibition of oxidative stress may maintain channel expression; targeting of specific modification sites (e.g., K1479 acetylation, Ser571 phosphorylation) or development of SUMOization/methylation modulators may correct channel dysfunction. In addition, intervention strategies targeting NAD+ metabolic imbalances (e.g., NAD+ precursors, PKCδ inhibitors) and lipid peroxidation inhibitors may be novel therapeutic directions for anti-ischemic arrhythmias.

## 5. Potential Pharmacologic Therapy

### 5.1 Sodium Channel Blockers

The classical class I antiarrhythmic drugs known as sodium channel blockers, and their subclasses Ia, Ib, and Ic, produce moderate, weak, or marked blockade of sodium channels and reduce AP phase 0 slope and overshoot while increasing, shortening, or preserving APD and the effective retention of the effective response period (ERP). Class Ia drugs such as quinidine, procainamide, and propyzamide bind preferentially to the open state of Nav1.5, blocking sodium channel opening and prolong the AP and ERP. In addition, these drugs block K^+^ channels and synergistically prolonging the APD. Prolongation of APD and QT interval by blocking sodium and potassium channels, however, may lead to EAD and induce tip-twist ventricular tachycardia. In addition, pharmacologic slowing of conduction velocity and inhomogeneity of the off-loading period can increase the risk of formation of foldback loops and trigger foldback arrhythmias, especially in the presence of QT prolongation, hypokalemia, or low heart rate. Ib analogs like lidocaine and mesylate bind preferentially to the Nav1.5 inactivated state and significantly inhibit the INaL, shortening the APD and ERP and eliminating the foldback. INa inhibition is enhanced in pathological conditions such as myocardial ischemia. It reduces the incidence of VAs among patients with an MI, but increases mortality. Therefore it is not recommended for prophylaxis. However, in patients with an MI who have developed VAs, lidocaine improves survival [76]. Mexiletine has also seen great advances in the treatment of LQTS. Mexiletine improves QTc, attenuates QT-RR slope, and eliminates 2:1 AV block and T-wave alternans by inhibiting late INa in patients with Timothy syndrome (TS) and in a TS model. This Id-like effect of mesylate suggests that it may be used as an antiarrythmic drug in clinical practice. The arrhythmogenic effects of class Ib drugs are relatively low, but there is a risk of excessive inhibition of sodium channels at too high a dose. Class Ic drugs such as propafenone and flutamide bind similarly to inactivated Nav1.5, from which it dissociates more slowly, and this impairs AP initiation and conduction and its antiarrhythmic effects. It was found that flutamide produces proarrhythmic effects in *SCN5A*+/- mice, MI, and BrS [77]. In patients with structural heart disease (e.g., post-MI, HF), class IC drugs significantly increase the risk of fatal arrhythmias.

### 5.2 Late Sodium Current Inhibitor

A modern classification of antiarrhythmic drugs was proposed in 2018, adding class Id late sodium current inhibitors to the original list. Inhibition of late sodium currents by class Id antiarrhythmic drugs such as ranolazine, GS458967, and F15845 have potential antiarrhythmic effects during cardiac ischemia INaL-related arrhythmias. Ranolazine is currently approved as an antianginal drug, and its perfusion significantly inhibits focal activity in the ischemic marginal zone of rabbit ischemic hearts, promotes VT/VF termination, improves ejection fraction, cardiac output, and wall motion abnormalities after reperfusion, and reduces myocardial necrosis [78]. F15845 selectively blocks sustained sodium current and is valuable in the treatment of ischemic arrhythmias [79]. GS458967 is antiarrhythmic in and ischemia-induced arrhythmia model in rabbits and is effective in protecting against both atrial and ventricular depolarization and repolarization abnormalities, and it is more effective than flucloxacillin or ranolazine in reducing INaL and arrhythmia [80]. Eleclazine (GS-6615, Dihydrobenzoxazepinone) is a novel, potent and selective late-stage INa inhibitor currently in clinical development that is up to 42 times more potent than ranolazine in reducing ischemic load *in vivo* [81]. Selective inhibition of late cardiac INa by Elecazine has a dual protective effect, preventing susceptibility to ischemic atrial fibrillation and reducing atrial and ventricular repolarization abnormalities before and during adrenergic stimulation without negative inotropic effects. Compound F15741 is also a selective and potent late current inhibitor, the application of which was found to reduce infarct size in porcine hearts, thus extending the therapeutic potential of late sodium current blockers during cardiac ischemia [82]. Therapeutic concentrations of late sodium current inhibitors have little or no effect on peak sodium current and/or IKr, and therefore have no or little pro-arrhythmic risk compared to classical class I or III antiarrhythmic drugs, especially in patients with ischemic heart disease. Side effects such as prolongation of the QT interval, gastrointestinal reactions, dizziness and headache, however, are also associated with late sodium current inhibitors. Therefore in patients with hepatic or renal insufficiency or coadministration of medications that prolong the QT interval, one needs to be vigilant about the risk of side effects, and to carefully monitor the QT interval and hepatic and renal function. Triiodothyronine (T3) [83], the anesthetic agent ketamine [84], and multiple ion channel blocker curcumin (Cur) [85] can exert cardioprotective effects after I/R by inhibiting late sodium currents. Uncovering the specific mechanisms as well as discovering new and more potent inhibitors of late sodium currents with fewer side effects will be the subject of future research.

### 5.3 Non-Classical Antiarrhythmic Drugs

Several other well known drugs that have shown antiarrhythmic properties are worth exploring. Statins can reduce cardiovascular disease mortality by lowering cholesterol levels, and they also show lipid-independent pleiotropic effects, many of which are cardioprotective. Statins with beneficial protective effects against life-threatening VAs have been studied. Simvastatin ameliorates I/R-induced reduction of INa in ventricular myocytes. Nie *et al*. [86] found that atorvastatin affects the INa of I/R-mimicking cells in the left ventricle of normal rats by directly blocking sodium channels. These non- cholesterol lowering effects demonstrate the importance of the pleiotropic effects of statins in the prevention and treatment of arrhythmias after myocardial ischemia. PUFAs also exert antiarrhythmic effects through various mechanisms involving anti-inflammation, reduction of Ca^2+^ overload and alteration of ion channel activity [87]. During periods of ischemia or I/R, DHA and EPA are able to block INa and INaL to exert antiarrhythmic effects [88]. But PUFAs’ antiarrhythmic potential has not yet been determined [87]. Activation of the renin-angiotensin system may also play a role in the treatment of cardiac arrhythmias. Angiotensin II (Ang II) induces VAs during I/R injury. As such, angiotensin-converting enzyme inhibitors/angiotensin receptor blockers also serve as potential antiarrhythmic agents. It has been shown that angiotensin-(1-7) (Ang-(1-7)) counteracts the effects of Ang II, significantly increases INa density, contributes to improved atrial conduction and reduces the potential for AF [89]. The continuation of in-depth research into the possible mechanisms behind these non-traditional antiarrhythmic drugs is essential since some studies have found less favorable results with these drugs [87]. Chinese medicinal preparations have also been used for the treatment of arrhythmias after cardiac ischemia. Sanwei sandalwood decoction (SWTX) significantly shifted the activation curve of Nav1.5 leftward by inhibiting Ito and Ikr, prolonging ventricular conduction, cardiac conduction dispersion and time course, and had a potential cardiac protective effect on myocardial I/R injury in rats [90]. The anti-arrhythmic Chinese medicine Wenxin Keli can shorten the QT interval and slow the heart rate by down-regulating *SCN5A* and ADRB2 in MI and up-regulating CHRM2, thus producing anti-arrhythmic effects [91]. Its active ingredient, lauric acid, is a potential novel INa blocker that promotes the antiarrhythmic effect of Wenxin Keli [92]. Paeonol, a representative active ingredient of peony bark, inhibits H/R-induced reduction of Nav1.5 and Kir2.1 current levels in H9c2 cells and reduces VAs in rats with an MI [93]. Chinese medicine emphasizes overall regulation and evidence-based treatment, improving myocardial electrical activity through multi-targeting, antioxidant, anti-inflammatory effects. It is suitable for chronic arrhythmias or as an adjunctive therapy, with relatively few side effects and suitable for long-term use. Modern drugs, on the other hand, are characterized by precise treatment, rapidly controlling arrhythmia by regulating specific ion channels, and are suitable for patients with acute episodes or acute and severe illnesses, but long-term use may cause side effects, such as arrhythmias. Traditional Chinese medicine and modern drugs can complement each other, and when used in combination, they can improve therapeutic effects and minimize side effects. During an AMI, mild hypothermia, a new antiarrhythmic resuscitation strategy, protects gap junction coupling and sodium channel function, attenuates conduction slowing and prevents conduction blocks [94]. The strategy for the treatment of arrhythmias after cardiac ischemia leaves many areas to be explored in addition to the classical antiarrhythmic drugs.

The application of some drugs exacerbates arrhythmias in patients with an MI. Chronic doses of macrolide antibiotics have potentially cardiotoxic effects in rats with an MI, being able to down-regulate the Nav1.5 channel, which is manifested by abnormal electrocardiogram alterations and pathologic manifestations [95]. Fluroquinolone antibiotics resulted in greater electrocardiographic disturbances and increased cardiac enzymes in rats with a history of AMI compared with non-infarcted rats, and their arrhythmogenic effects were associated with improved expression of the cardiac ion channels Kv4.3 and Nav1.5 [96]. Nonsteroidal Anti-inflammatory Drugs (NSAIDs) can also cause certain cardiac side effects, in the form of MIs and arrhythmias. A study has found a potential cardiac risk associated with their use in their inhibitory effects on Nav1.5 and Kv11.1 [97]. Therefore patients with a history of an MI should be carefully medicated and closely monitored.

## 6. Conclusions

Nav1.5, a core component of the cardiac voltage-gated sodium channel, plays a key role in arrhythmogenesis and development after an MI. Its regulatory mechanisms are extremely complex, including genetic polymorphisms of *SCN5A*/*SCN10A*, transcriptional and post-transcriptional regulation, translational and post-translational modifications, and protein degradation and transport, which together affect the dynamic balance of the peak sodium currents, INa and INaL, and consequently the electrophysiological stability of the heart. Abnormalities in Nav1.5 expression and function can lead to slowed cardiac conduction, prolonged action potentials, and decreased repolarization reserve, which in turn induce severe VAs and even SCD. Nav1.5-based precision modulation strategies are emerging as a potential source for antiarrhythmic therapy. Class I antiarrhythmic drugs (e.g., lidocaine, mesylate) and class Id late sodium current inhibitors (e.g., ranolazine) show good clinical potential in reducing INaL and improving arrhythmias. Non-classical antiarrhythmic therapies such as statins, PUFAs, and Chinese herbal medicines (e.g., Wenxin Keli) are gradually being introduced into clinical practice. The development of novel INaL inhibitors with more selectivity and fewer side effects than ranolazine as well as the exploration of new antiarrhythmic agents is will be the subject of future research. Individualized therapy remains an important direction for future development. Since there are significant differences in drug response among SNPs in *SCN5A*/*SCN10A*, patient genotype-based precision therapy strategies are expected to optimize the efficacy and safety of antiarrhythmic drugs. Signaling pathways such as Wnt/β-catenin, MAPK, NAD+ metabolism, and the ubiquitin-proteasome system are all involved in the regulation of Nav1.5. Therefore targeting interventions of these pathways, such as EZH2 inhibitors, Sirt1 activators, and Nedd4-2 modulators, will be the subject of future studies in an attempt to develop new therapeutic strategies for the treatment of arrhythmias in clinical practice.
